# Synthesis and Cytotoxic Evaluation of Novel 3-Substituted Derivatives of 2-Indolinone 

**Published:** 2012

**Authors:** Shaya Mokhtari, Mahmoud Mosaddegh, Maryam Hamzeloo Moghadam, Zohreh Soleymani, Saeideh Ghafari, Farzad Kobarfard

**Affiliations:** a *Department of Medicinal Chemistry, School of Pharmacy, Shahid Beheshti University of Medical Sciences, Tehran, Iran.*; b *Traditional Medicine and Materia Medica Research Center, Shahid Beheshti University of Medical Sciences, Tehran, Iran.*; c *Pharmaceutical Research Sciences Center, Shahid Beheshti University of Medical Sciences, Tehran, Iran. *; d *Student Research Committee, School of Pharmacy, Shahid Beheshti University of Medical Sciences, Tehran, Iran.*; e *Phytochemistry Research Center, Shahid Beheshti University of Medical Sciences, Tehran, Iran*

**Keywords:** 3- substituted derivatives of 2-indolinones, Indole derivatives, Cytotoxicity, Colon and breast cancer, MTT assay

## Abstract

The assessment of the degree or rate of cellular proliferation and cell viability is critical for the assessment of the effects of drugs on both normal and malignant cell populations. In the present study, a few novel 3-substituted derivatives of 2-indolinones were synthesized by condensation of substituted oxindole or isatin derivatives with appropriate aldehydes or primary aromatic amines respectively. The synthesized compounds were screened for their cytotoxicity against HT-29 (human colon adenocarcinoma cell line) and MCF-7 (human breast adenocarcinoma cell line) cells using short term cytotoxicity MTT assay protocol. A few derivatives with IC_50_ < 10 µM were identified among them. he compound bearing 5-bromo substitution was the most potent derivative. Global physicochemical properties for compounds IVa-e and Va-h were calculated and the two most active compounds (IVa and IVb) showed similar CLogP values.

## Introduction

Cancer is a major health concern all around the world. Progresses in prevention and treatment of cancer have decreased the health rate, but the number of new diagnoses continues to increase. Therefore, new and more efficient anticancer agents are required to battle different cancer diseases. As a part of our efforts to find new chemotherapeutic agents as potential anticancer agent, we have synthesized a series of indole derivatives. Indole derivatives represent many important classes of therapeutical agents in medicinal chemistry such as anti cancer (1), antioxidant (2), anti rheumatoid arthritis (3) and anti HIV (4, 5).

Some studies revealed that a group of 2-phenyl indole sulfamat are steroid sulfatase inhibitors with anti proliferative activity in breast cancer cells (6).

Some of the sulfur containing 2-phenyl indole analogues show *in-vivo* antineoplastic and anti estrogenic activity (7, 8).

**Figure 1:**
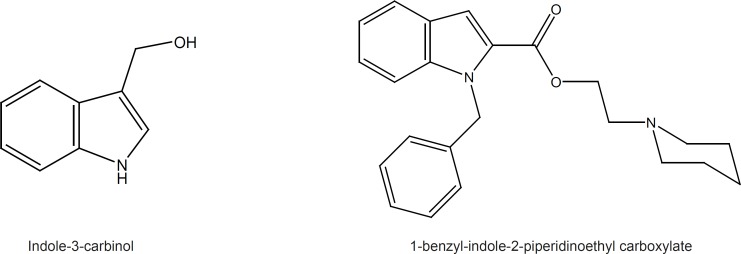
Two biologically effective indole structures

The chemopreventive properties of cruciferus vegetables are attributed to the antitumor activity of indole-3-carbinol (Figure 1) and its metabolic derivatives, which have shown great potential for both prevention and treatment of cancer through numerous mechanisms such as induction of apoptosis and cell cycle arrest, antiestrogenic activity, gene expression modulation and prevention of carcinogen-DNA adduct formation (9,10). 

Olgen *et al.* have viewed indole ring as heterocyclic ATP analogue and discovered a few new indole derivatives with tyrosine kinase inhibitory activity. They have reported that 1-benzyl-indole-2-piperidinoethyl carboxylate is a potent inhibitor of Src tyrosine kinase with IC_50_ of 1.34 µM (11) (Figure 1).

**Figure 2:**
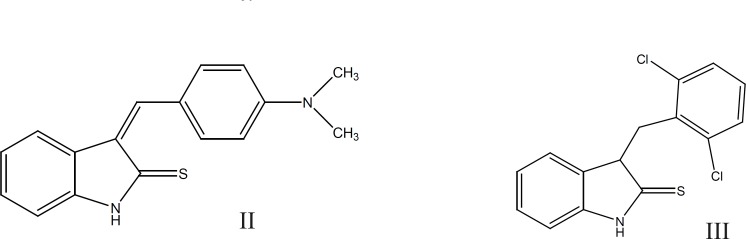
Chemical structure of (II) and (III) which are moderately active Src PTK inhibitor

They also introduced a series of 3-(substituted-benzylidene)-1,3-dihydro-indolin-2-thione derivatives and their corresponding indolin-2-one congeners and evaluated their ability to inhibit Src PTK. In this study, (Z)-3-(4`-dimethylaminobenzylidene)-1, 3-dihydro-indolin-2-thion (II) and (Z)-3-(2`, 6`-dichlorobenzylidene)-1,3dihydro-indolin-2-thion (III), were identified as moderately active Src PTK inhibitors with IC_50_ of 21.91 and 21.20µM respectively (12) (Figure 2).

In an effort to find novel indole-based compounds with potential anticancer activity, a few 3-benzylidene indole-2-one and 3-phenylimino indole-2-one derivatives were synthesized and evaluated for their cytotoxic activity against HT-29 (human colon adenocarcinoma cell line) and MCF7 (human breast adenocarcinoma cell line) using short term cytotoxicity MTT assay protocol. It is proven that thyrosine kinases of the Src family (SFK) are frequently deregulated in human colorectal cancer (CRC) (13).The overexpression of thyrosine kinases in high percentages in human breast cancers is also well documented (14).

A series of thirteen 3-benzylidene indole-2-ones and 3-phenyliminoindole-2-ones (IV_a-e_ and V_a-h_) were prepared in our lab as shown in (Table 1). These compounds were screened for their cytotoxic activities against colon (HT29) and breast (MCF7) cancer cell lines.

**Figure 3:**
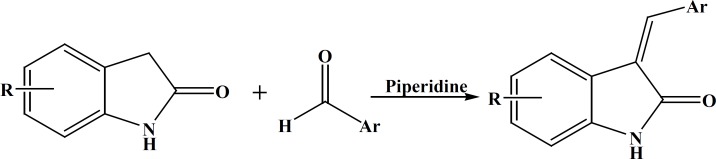
Compounds IV_a-e_ synthesis scheme

Compounds IV_a-e_ were synthesized by condensation of appropriate indole-2-one with different aromatic aldehydes in the presence of piperidine as base (Figure 3).

**Figure 4:**
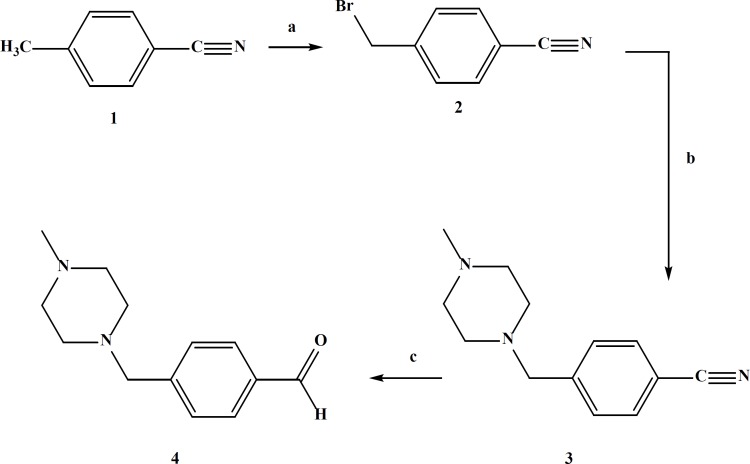
Synthesis of 4-((4-methylpiperazin-1-yl) methyl)benzaldehyde (4); (a)Dibenzoyl peroxide, NBS, CCl_4_, reflux, 24 h; (b) 4-methylpiperazine, CHCl_3_, 24 h; (c) Raney Nickel alloy, formic acid 75%, reflux, 2 h

In the case of compound IV_e _the aldehyde was synthesized in three steps starting from 4-(bromomethyl)benzonitrile (Figure 4).

**Figure 5:**
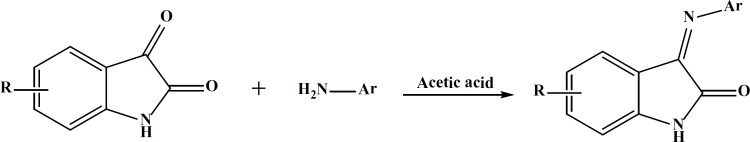
Compounds V_a-h_ synthesis scheme

Compounds V_a-h _were synthesized by condensation of the appropriate isatin derivatives with the proper aromatic amines in the presence of acetic acid (Figure 5).

## Experimental

*Chemistry*

All solvents, reagents and catalysts were of analytical grade and used without further purification. The melting points (°C) were determined by open capillary method on an Electrothermal melting point apparatus and were uncorrected. The purity of compounds was confirmed by thin layer chromatography using Whatman Sil G/UV_254_ silica gel plates as the stationary phase and with suitable mobile phase with fluorescent indicator, and the spots were visualized under 254 and 366 nm illumination. Infrared spectra were recorded as thin films on KBr plates with υ_max _in inverse centimeters. ^1^H NMR spectra were recorded on a Bruker DRX**-**Avance (500 MHz) and or (250 MHz) spectrometer using DMSO-d6 and CDCl_3 _as solvents and Chemical shift values are expressed in ppm (parts per million) relative to tetramethylsilane (TMS) as internal standard; s = singlet, d = doublet, dd = double doublet, t = triplet, q = quartet, m = multiplet, brs = broad singlet. Mass analyses were performed with an Agilent 6400 Series equipped with an electrospray ionization source (capillary voltage at 4000V, nebulizing gas temperature at 300 °C, nebulizing gas flow at 12 L/ min ) . All the compounds were analyzed for C, H, N, and S on a Costech model 4010 and agreed with the proposed structures within ± 0.4% of the theoretical values.

*General procedure for synthesis of 3-(Substituted benzylidenyl)-indolin-2-one analogues (Compounds IV*_a-e_ *)*

A reaction mixture of the proper oxindole (1 equiv), aldehyde (1.2 equiv), and piperidine (0.1 equiv) in ethanol (1-2 mL/1 μmol oxindole) was stirred at 90 °C for 3-5 h (15). After the mixture cooled, the precipitate was filtered, washed with cold ethanol and hexane and recrystallized from suitable solvent to give the target compound.

*4-((5-bromo-2-oxoindolin-3-ylidene)methyl)benzonitrile* *(IV*_a_*)*

Yield 58%, mp 267-272.5 °C (dec.); ethanol; IR (KBr) υ_max _3195 (NH), 2246(nitrile), 1712 (C=O), 1613 cm^-1^; ^1^H NMR (250 MHz, DMSO-*d*6) *δ *10.86 (s,1H, NH),8.4 (d, 2H , *J=* 8.25 ; H-3`,5`),7.9 (m), 7.71 (s,1H, H-vinyl), 7.4 (m), 6.83 (dd, 2H, *J=15, 8.2*5; H-2`,6`), mixture of Z and E isomers ; ^13^C-NMR (62.9 MHz, DMSO-*d*6) *δ* 116.2, 116.7, 116.9, 117.5, 117.9, 123.2, 123.4, 127.1, 127.9, 129.5, 131.2, 133.1, 133.3, 134.6, 136.6, 136.8, 137.3,137.8, 140, 140.7, 142.6, 143.6, 145, 147.1, 171.1, 172.4 ; ESI-MS: Observed ( M+H^+^ )= 325, 327 (M+Na^+^) = 347, 349. Calcd for C_16_H_9_BrN_2_O = 325.16; Anal. Found: C, 59.21; H, 2.78; Br, 24.59; N, 8.60; O, 4.91. Calcd for C_16_H_9_BrN_2_O: C, 59.10; H, 2.79; Br, 24.57; N, 8.62; O, 4.92%.

*N-(2-fluoro-4-((2-oxoin dolin-3-ylidene)* *methyl)phenyl)acetamide (IV*_b_ *)*

Yield 15%, mp 249-252 ºC (dec.); ethanol; IR (KBr) υ_max _3185 (NH), 3175(NH of acetamide), 1710 (C=O), 1660(C=O of acetamide), 1613 cm^-1^; ^1^H NMR (250 MHz, DMSO-*d*6) *δ *10.69 (s, 1H, NH-1), 10 (s, 1H, NH of acetamide), 8.14 (dt, 1H, *J= *8.5, 1.75; H-6), 7.72(m), 7.23(dd, 2H, *J=*15,7.5; H-5`, 6`), 6.9 (m), 2.14 (s, 3H, NHCOCH_3_-4`), mixture of Z and E isomers; ^13^C-NMR (62.9 MHz, DMSO-*d*6) *δ* 109.3, 110.1, 116.2, 116.5, 117.5, 117.8, 118.6, 119.6, 120.6, 121.1, 121.2, 122.3, 123, 124, 125.6, 126.3, 127.4, 127.5, 127.7, 128.9, 129.5, 129.7, 130.2, 130.4, 130.5, 130.7, 134.2, 140.6, 142.9, 150.5, 154.4, 167.2, 168.5, 169, 169.1; Anal. Found: C, 68.93; H, 4.41; F, 6.43; N, 9.47; O, 10.82. Calcd for C_17_H_13_FN_2_O_2_ : C, 68.91; H, 4.42; F, 6.41; N, 9.45; O, 10.80%.

*N-(2-chloro-4-((2-oxoindolin-3-ylidene)methyl)phenyl) acetamide* *(IV*_c_*)*

Yield 56%, mp 220-228 °C (dec.); ethanol; IR (KBr) υ_max _3184 (N-H), 3082 (NH of acetamide), 1702 (C=O), 1662(C=O of acetamide), 1611 cm^-1^;^ 1^H NMR (250 MHz, DMSO-*d*6) *δ *10.65 (s, 1H, NH-1), 9.66 (s, 1H, NH of acetamide), 7 (m), 2.15 (s, 3H, NHCOCH_3_ -4`), mixture of Z and E isomers; ^13^C-NMR (62.9 MHz, DMSO-*d*6) *δ* 23.5, 23.6, 109.4, 110.2, 119.8, 120.6, 121.1, 121.2, 122.2, 124, 124.4, 124.6, 125, 125.4, 126.8, 127.8, 128.2, 129.1, 130.2, 130.3, 131.2, 131.5, 131.7, 132.1, 133.8, 134.6, 136, 136.5, 140.7, 142.9, 167.1, 168.5, 169; Anal. Found: C, 65.31; H, 4.2; Cl, 11.36; N, 8.95; O, 10.21. Calcd for C_17_H_13_ClN_2_O_2_: C, 65.29; H, 4.19; Cl, 11.34; N, 8.96; O, 10.23%

*Preparation of 4-(bromomethyl)benzonitrile(* 2 *)*

4-toluenitrile (1) (0.1 mol) was added to a flask containing N-bromosuccinimide (0.11 mol) and dibenzoyl peroxide (500 mg) in dried carbon tetrachloride (200 mL).The reaction mixture was refluxed under nitrogen atmosphere overnight. Then the mixture cooled and filtered and the filtrate was concentrated and 300 mL hexane was added to this solution to form the white crystals of 4-(bromomethyl) benzonitrile (16). The product was purified by recrystallization from chloroform. The yield was (50%), mp = 115-117 °C.

*Preparation of 4-((4-methylpiperazin-1-yl)methyl)benzonitrile(* 3 *)*

1-(Bromo) toluenitrile (10.2mmol) in 20 mL of chloroform was stirred at room temperature before dropwise addition of a solution of 1-methyl piperazine (28 mmol) in 5 mL chloroform. The reaction mixture was stirred at room temperature for 24 h then the reaction was quenched with water and further stirred for 30 min before extracting with chloroform. The organic layer was dried and concentrated (17). In the residue were formed crystals which were washed with hexane. It was pure 4-((4-methylpiperazin-1-yl) methyl) benzonitrile; yield (35%), mp = 65-67°C (Ref: 62-64°C); ESI-MS: Observed (M+H^+^) = 216. Calcd for C_13_H_17_N_3_ = 215.2.

*Preparation of 4-((4-methylpiperazin-1-yl) methyl)benzaldehyde (* 4 *) (* 18 *)*

4-((4-methylpiperazin-1-yl)methyl)benzonitrile (9 mmol)was dissolved in formic acid 75% (37 mL) and raney nickel alloy (2 g) was added to this solution. The mixture was refluxed for 2 h then filtered with celite and washed with 20 mL of cold ethanol 96°.The filtrate was concentrated and again filtered to remove the green colloidal impurities to give (1.8 g) crude product, ESI-MS: Observed (M+H^+^) = 219. Calcd for C_13_H_18_N_2_O = 218.29

*Synthesis of 3-(4-((4-methylpiperazin-1-yl)methyl)benzylidene)indolin-2-one (IV*_d_ *)*

A reaction mixture of oxindole (1 equiv), 4-((4-methylpiperazin-1-yl) methyl)benzaldehyde (1.2 equiv), and piperidine (0.1 equiv) in ethanol (1-2 mL /1 *μ*mol oxindole) was stirred at 90 °C overnight. The solvent of the reaction mixture was evaporated and the residue was dissolved in warm ethyl acetate and passed through a column of silica gel. The polarity of eluting solvent was increased with the addition of methanol to the ethyl acetate. The yellow liquid phase was collected and the solvent was evaporated to achieve 3-(4-((4-methylpiperazin-1-yl)methyl)benzylidene) indolin-2-one**: **Yield 38%, mp 264-269 °C (dec.) ; ethanol; ^1^H NMR (500 MHz, DMSO-*d*6) *δ *10.58 (s, 1H, NH-1), 7.66 (d, 2H, *J*= 8, H-2′,6′), 7.59 (s , 1H, H-vinyl),7.56(d, *J*= 8, 1H, H-4), 7.43(d, 2H, *J*= 8, H-3‹, 5`), 7.21(t, 1H, *J*= 7.5, H-6), 6.85 (m, 2H, H-5,7),3.52 (s, 2H, CH_2_), 2.37(m, 8H, CH_2_ piperazine), 2.16(s, 3H, CH_3_) ; ^13^C-NMR (62.9 MHz, DMSO-*d*6) *δ* 45.2, 52.1, 54.3, 61.5, 110.1, 120.8, 121.1, 122.3, 127.1, 128.5, 129, 129.2, 130, 131.8, 132.9, 135.7, 140.2, 142.8, 164.6, 168.6; ESI-MS: Observed ( M+H^+^ ) = 334. Calcd for C_21_H_23_N_3_O = 333.43; Anal. Found: C, 75.70; H, 6.93; N, 12.58; O, 4.78.Calcd for C_21_H_23_N_3_O: C, 75.65; H, 6.95; N, 12.60; O, 4.80%.

*3-((5-(4-fluorophenyl)pyridin-3-yl)methylene)indolin-2-one* *(IV*_e_*)*

Yield 85%, mp 204-206.9 ºC (dec.); ethanol; IR (KBr) υ_max _3160 (N-H), 1720 (C=O), 1689, 1607 cm^-1^; ^1^H NMR (250 MHz, DMSO-*d*6) *δ *10.70 (s, 1H, NH), 9.23 (t,1H, *J= *2; H-2′), 9.15 (d,1H,* J*=1.75; H-6′),8.9 (m,1H, H-4′), 7.91 (s, 1H, H-vinyl), 7.84 (m), 7.72 (t, 1H, *J *= 7.5 Hz; H-4), 7.39 (m), 7.26 (t, 1H,* J *=7.5; H-5), 7.05 (t,1H, *J *=7.5; H-6), 6.87 (m,1H; H-7) , mixture of Z and E isomers ; ^13^C-NMR (62.9 MHz, DMSO-*d*6) *δ* 115.9, 116.2, 120.2, 120.7, 121.3, 122.1, 124.3, 128.9, 129, 129.1, 129.6, 129.8, 130.6, 130.7, 131.8, 132.4, 132.7, 132.8, 133.3, 133.7, 134.3, 135.6, 141.1, 143.2, 147.9, 148, 148.1, 150.9, 160.5, 164.4, 167, 168.1; ESI-MS: Observed ( M+H^+^ ) = 317. Calcd for C_20_H_13_FN_2_O = 316.3. Anal. Found: C, 75.96; H, 4.15; F, 6.03; N, 8.84; O, 5.05. Calcd for C_20_H_13_FN_2_O: C, 75.94; H, 4.14; F, 6.01; N, 8.86; O, 5.06%.

**Table1:** Chemical structute of synthesized 3-benzylidene indole-2-ones (IV_a-e_) and 3- phenyliminoindole-2-ones (V_a-h_).

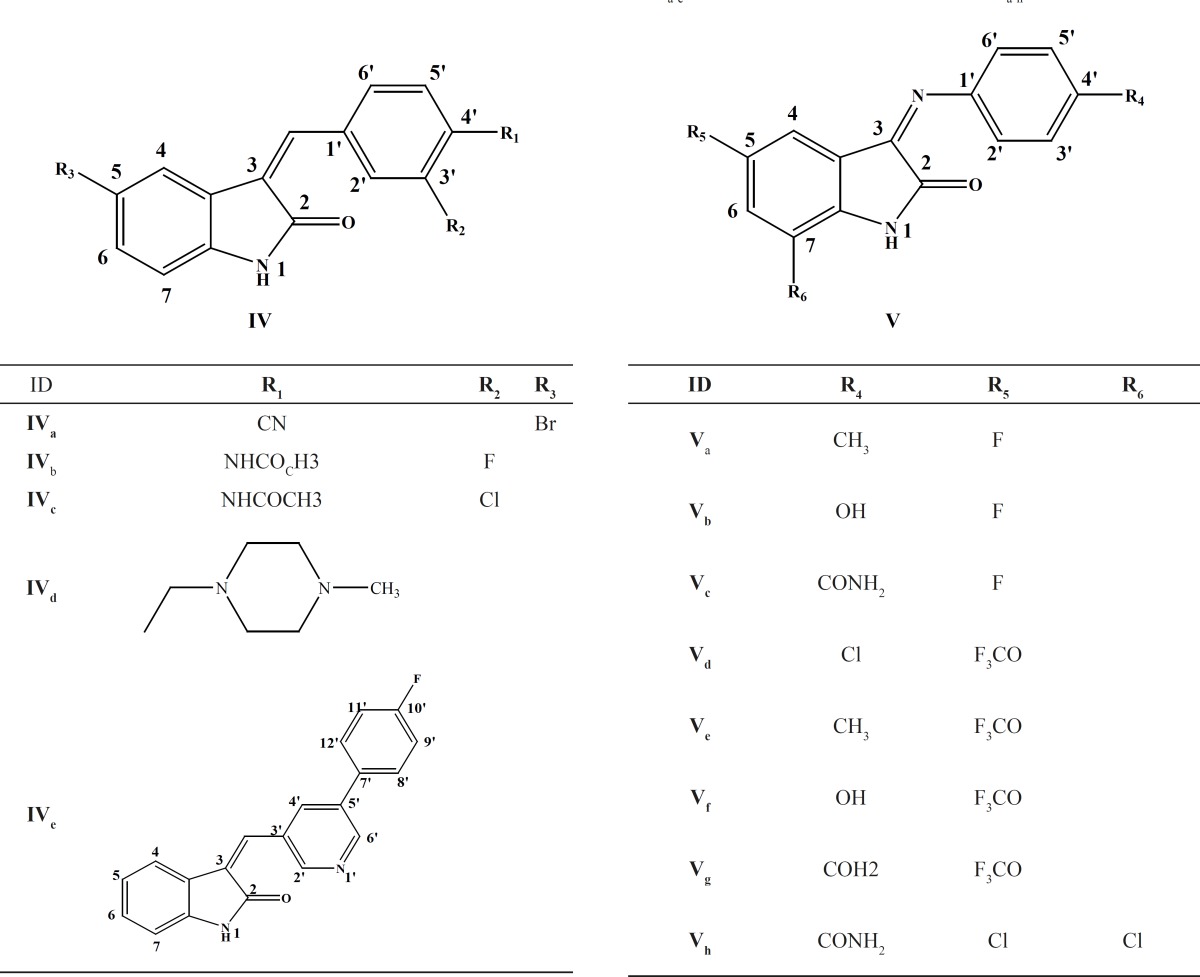

*General Procedure for Compounds (V*_a-h_ *)*

A mixture of indole-2, 3-dione (0.01M) and Amine (0.01M) in absolute ethanol(20 mL) was refluxed for 20 h in the presence of 2-3 drops of glacial acetic acid (19). After cooling, the resultant was filtered and washed with hexane and recrystallised from appropriate solvent to give compounds V_a-h_.

*5-fluoro-3-(p-tolylimino)indolin-2-one (V*_a_ *)*

Yield 38%, mp 264-269 ºC (dec.) ; ethanol; IR (KBr) υ_max _3256 (N-H), 1741 (C=O),1652 (C=N) ,1613 cm^-1^; ^1^H NMR (250 MHz, DMSO-*d*6) *δ *11.03 (s,1H, NH-1), 7.15 (m, 6H, H-6,7,2′,3′,5′,6′), 6.14 (dd, 1H, *J *= 8.5, 2.5 Hz; H-4), 2.37(s, 3H, CH_3_-4′), mixture of Z and E isomers; ^13^C-NMR (62.9 MHz, DMSO-*d*6) *δ* 20.5, 111.5, 111.9, 112.5, 112.6, 115.9, 116, 117.4, 119.8, 120.5, 120.9, 128.7, 130, 134.6, 143.2, 147.2, 154.3, 154.8, 158.5, 163.5; ESI-MS: Observed (M+H^+^) = 255 Calcd for C_15_H_11_FN_2_O = 254.2;Anal. Found: C, 70.89; H, 4.31; F, 7.45; N, 11.03; O, 6.27. Calcd for C_15_H_11_FN_2_O: C, 70.86; H, 4.36; F, 7.47; N, 11.02; O, 6.29%.

*5-fluoro-3-(4-hydroxyphenylimino)indolin-2-one (V*_b_ *)*

Yield 74%, mp 307-320 ºC (dec.) ; ethanol; IR (KBr) υ_max _3298, 1711 (C=O), 1619, 1599 (C=N) cm^-1^; ^1^H NMR (250 MHz, DMSO-*d*6) *δ *10.97 (s,1H, NH-1), 9.66 (s, 1H, OH-4′), 7.26 (m),6.84 (m), 6.44 (dd,1H, *J *=8.5, 2.5 Hz; H-4), mixture of Z and E isomers ; ^13^C-NMR (62.9 MHz, DMSO-*d*6) *δ* 108.6, 109, 111.1, 111.3, 111.5, 112.3, 112.4, 114.7, 115.9, 116, 116.2, 119, 119.4, 119.9, 120.1, 120.5,124.6, 138.8, 140.7, 141, 142.9, 143, 153.4, 154.8, 155.8, 156.9, 158.6, 158.9, 163.7; ESI-MS: Observed ( M+H^+^ ) = 257. Calcd for C_14_H_9_FN_2_O_2_ = 256.23; Anal. Found: C, 65.68; H, 3.53; F, 7.42; N, 10.91; O, 12.47.Calcd for C_14_H_9_FN_2_O_2_: C, 65.62; H, 3.54; F, 7.41; N, 10.93; O, 12.49%.

*4-(5-fluoro-2-oxoindolin-3-ylideneamino)benzamide* *(V*_c_*)*

Yield 19%, mp 277-310 °C (dec.) ; ethanol; IR (KBr) υ_max _3394 (NH_2_), 3232 (N-H), 1741 (C=O Oxindole),1673 (C=O benzamide), 1630 (C=N) cm^-1^; ^1^H NMR (250 MHz, DMSO-*d*6) *δ *11.09 (s,1H, NH-1), 7.94(m), 7.33 (m),7.06 (m), 6.92 (m), 5.97(dd, 1H,* J=* 8.25, 2.5; H-4), mixture of Z and E isomers; ^13^C-NMR (62.9 MHz, DMSO-*d*6) *δ* 109.7, 110.1, 111.7, 112.1, 112.7, 112.8, 115.8, 115.9, 116.9, 118.2, 120.9, 121.3, 127.9, 129.2, 130.8, 143.4, 152.4, 163.3, 167.3, 167.6 ; ESI-MS: Observed ( M+H^+^ ) = 284. Calcd for C_15_H_10_FN_3_O_2_ = 283.2; Anal. Found: C, 63.70; H, 3.55; F, 6.72; N, 14.85; O, 11.33. Calcd for C_15_H_10_FN_3_O_2_ : C, 63.60; H, 3.56; F, 6.71; N, 14.83; O, 11.30%.

*3-(4-chlorophenylimino)-5-(trifluoromethoxy)indolin-2-one (V*_d_ *)*

Yield 43%, mp 230-240 ºC (dec.); ethanol; IR (KBr) υ_max _3282 (N-H), 1746 (C=O),1628 (C=N) cm^-1^;^ 1^H NMR (250 MHz, DMSO-*d*6) *δ *11.21 (s,1H, NH-1), 7.46 (m), 7.06 (m), 6.20 (d,1H, *J=* 1.25 Hz; H-4), mixture of Z and E isomers ; ^13^C-NMR (62.9 MHz, DMSO-*d*6) *δ* 112, 112.7, 116, 117.8, 119.3, 121.1, 127.3, 127.4, 128.2, 129.4, 129.5, 142.1, 143.3, 144.7, 146, 147.3, 148.7, 152.9, 154.8, 158.4, 163.3. 6 ; ESI-MS: Observed ( M+H^+^ ) = 342. Calcd for C_15_H_8_ClF_3_N_2_O_2_ = 340.7; Anal. Found C, 52.92; H, 2.36; Cl, 10.43; F, 16.75; N, 8.21; O, 9.37. Calcd for C_15_H_8_ClF_3_N_2_O_2_ : C, 52.88; H, 2.37; Cl, 10.41; F, 16.73; N, 8.22; O, 9.39%.

*3-(p-tolylimino)-5-(trifluoromethoxy)indolin-2-one (V*_e_ *)*

Yield 59%, mp 266.5-268.5 ºC (with dec.); ethanol; IR (KBr) υ_max _3254 (N-H), 1741 (C=O),1619(C=N) cm^-1^;^ 1^H NMR (250 MHz, DMSO-*d*6) *δ *11.3 (s,1H, NH-1), 7.15(m, 6H, H- 6, 7, 2′, 3′, 5′, 6′), 6.25 (d, 1H, *J *= 1.25 Hz, H-4), 2.36 (s, 3H, CH_3_-4′), mixture of Z and E isomers; ^13^C-NMR (62.9 MHz, DMSO-*d*6) *δ* 20.41, 20.55, 111.8, 112.6, 116.1, 117.3, 117.8, 119.9, 127.1, 128.7, 129.9, 134.7, 142.1, 145.7, 147.4, 154.1, 163.4 ; ESI-MS: Observed ( M+H^+^ ) = 321.Calcd for C_16_H_11_F_3_N_2_O_2_ = 320.2; Anal. Found: C, 60.2; H, 3.45; F, 17.81; N, 8.74; O, 9.97. Calcd for C_16_H_11_F_3_N_2_O_2_ : C, 60.00; H, 3.46; F, 17.80; N, 8.75; O, 9.99%.

*3-(4-hydroxyphenylimino)-5-(trifluoromethoxy)indolin-2-one (V*_f_ *)*

Yield 80%, mp 230-238 °C (dec.); ethanol; IR (KBr) υ_max _3314 (O-H), 3209 (N-H), 1732 (C=O),1627 (C=N) cm^-1^;^1^H NMR (250 MHz, DMSO-*d*6) *δ *11.13 (1H,s, NH-1), 9.66 (s,1H, OH-4′), 7.37 (m), 6.89(m), 6.60 (d, 1H , *J *= 1.25 Hz; H-4), mixture of Z and E isomers; ^13^C-NMR (62.9 MHz, DMSO-*d*6) *δ* 111.5, 112.4, 114.7, 115.9, 116.3, 117.5, 119.9, 123.8, 124.8, 125.8, 126.8, 138.7, 141, 142.1, 143.4, 145.6, 149.1, 153.2, 155.9, 157.1, 158.9, 163.7; ESI-MS: Observed ( M+H^+^ ) = 323.Calcd for C_15_H_9_F_3_N_2_O_3_ = 322.2; Anal. Found: C, 55.93; H, 2.84; F, 17.67; N, 8.69; O, 14.92. Calcd for C_15_H_9_F_3_N_2_O_3_: C, 55.91; H, 2.82; F, 17.69; N, 8.69; O, 14.90%.

*4-(2-oxo-5-(trifluoromethoxy)indolin-3-ylideneamino)benzamide (V*_g_ *)*

Yield 49%, mp 296-304 °C (dec.); ethanol; IR (KBr) υ_max _3429 (NH_2_), 3121 (N-H), 1727 (C=O Oxindole),1697 (C=O benzamide), 1608 (C=N) cm^-1^ ;^1^H NMR (250 MHz, DMSO-*d*6) *δ *11.22 (brs,3H, NH-1, CONH_2_-4′), 7.48 (m,6H, H-6,7,2′, 3′, 5′, 6′), 6.10 (s,1H, H-4), mixture of Z and E isomers ; ^13^C-NMR (62.9 MHz, DMSO-*d*6) *δ* 112, 112.7, 116, 116.8, 118, 118.3, 121.8, 122.1, 127.4, 127.9, 129.1, 130.1, 130.8, 142.1, 143.3, 144.8, 145.9, 151.5, 152.5, 154.4, 158.4, 163.2, 167.1, 167.6; ESI-MS: Observed ( M+H^+^ ) = 350. Calcd for C_16_H_10_F_3_N_3_O_3_= 349.26; Anal. Found: C, 55.05; H, 2.88; F, 16.30; N, 12.05; O, 13.72. Calcd for C_16_H_10_F_3_N_3_O_3_: C, 55.02; H, 2.89; F, 16.32; N, 12.03; O, 13.74%.

*4-(5, 7-dichloro-2-oxoindolin-3-ylideneamino)benzamide (V*_h_ *)*

Yield 58%, mp 322-325 °C (dec.); ethanol; IR (KBr) υ_max _3460 (NH_2_), 3339 (N-H), 1733(C=O Oxindole), 1662(C=O benzamide),1610(C=N) cm^-1^; ^1^H NMR (250 MHz, DMSO-*d*6) *δ *11.65 (s,1H, NH-1), 11.51 (s,2H, CONH_2_-4′), 7.3 (m), 6.18 (d, 1H, *J *=1.75 Hz; H-4), mixture of Z and E isomers ; ^13^C-NMR (62.9 MHz, DMSO-*d*6) *δ* 112.4, 115.8, 116.6, 116.9, 117.9, 118.4, 120.6, 120.8, 121.3, 122.8, 123.3, 123.9, 125.6, 126.8, 127, 127.9, 129, 129.2, 130.3, 131, 133, 135, 142.2, 143.6, 146.5, 151.2, 151.6, 152, 153.4, 158.1,159.4, 163, 167.2, 167.5, 168; ESI-MS: Observed ( M+H^+^ ) = 335.Calcd for C_15_H_9_Cl_2_N_3_O_2_ = 334; Anal. Found: C, 53.91; H, 2.70; Cl, 21.24; N, 12.58; O, 9.59. Calcd for C_15_H_9_Cl_2_N_3_O_2_ : C, 53.91; H, 2.71; Cl, 21.22; N, 12.57; O, 9.58%.

**Table 2:** IC_50_ values of Compound IV_a-e _, V_a-h_ and Tamoxifen on MCF7 and HT29 cell lines by MTT assay

**Comp. ID.**	**MCF-7** ^a^		**HT29** ^b^	
	**IC**_50_ **(µM)**	**RSD%**	**IC**_50_ **(µM)**	**RSD%**
**IV**_a_	6	7.9	13.13	4.3
**IV**_b_	9.3	5.0	10.75	6.8
**IV**_c_	>100	-	38.27	4.3
**IV**_d_	42.07	3.8	7.51	6.9
**IV**_e_	8.54	6.7	>100	-
**V**_a_	>100	-	>100	-
**V**_b_	>100	-	>100	-
**V**_c_	>100	-	>100	-
**V**_d_	>100	-	90.31	2.1
**V**_e_	>100	-	>100	-
**V**_f_	>100	-	>100	-
**V**_g_	27.2	7.1	61.9	3.4
**V**_h_	65.86	8.1	>100	-
**TAMOXIFEN**	6.05	4.9	10.36	3.7

^a^ human breast adenocarcinoma cell line.^ b^ human colon adenocarcinoma cell line

*Cytotoxic activity*

*Cell lines*

HT-29(human colon adenocarcinoma cell line) and MCF-7 cells (human breast adenocarcinoma cell line*) *were used for the *in-vitro *screening of newly synthesized compounds. Cell lines were obtained from Pasture Institute of Iran, Tehran, Iran. Each cell line was cultured in suitable medium for desired growth, plus 10% FBS and 1% penicillin-streptomycine in a humidified incubator at 37°C in an atmosphere of 95% O2 and 5% CO2. Then the growth curve of each cell line was plotted.

*Subculture of adherent cell lines*

Cultures were observed using an inverted microscope to assess the degree of confluency and the absence of bacterial and fungal contaminants was confirmed. Cell monolayer was washed with PBS (Phosphate buffer solution) using a volume equivalent to half the volume of culture medium. Trypsin was added on to the washed cell monolayer, flask was rotated to cover monolayer with trypsin. Flask was returned to the incubator and left for 2-10 min. The cells were examined using an inverted microscope to ensure that all the cells were detached and floated. The cells were resuspended in a small volume of fresh serum containing appropriate medium.A 100 μL aliquot was removed to perform a cell count. The required number of cells were transferred to plates (tissue culture grade, 96 wells, round bottom) and incubated for 24 h at 37 °C and 5 % CO2.

**Table 3:** Global physicochemical properties for compounds IV_a-e_ and V_a-h_

**Compound**	**CLogP ** ^a^	**P** ^ b^	**V ** ^c^	**SA** ^d^	**DM** ^e^
**IV**_a_	3.7836	1057.02	81.58	330.762	4.144
**IV**_b_	3.7808	5454.61	92.26	123.949	2.146
**IV**_c_	2.0496	2139.00	81.36	300.439	4.293
**IV**_d_	2.3696	3488.20	85.56	118.548	3.918
**IV**_e_	3.538	5205.99	103.14	192.032	3.159
**V**_a_	3.292	926.57	71.75	571.2	1.158
**V**_b_	2.126	1049.3	67.67	133.9	2.338
**V**_c_	1.306	1166.7	75.06	249.2	5.4
**V**_d_	4.391	1648.15	78.9	235.2	2.44
**V**_e_	4.177	1725.83	80.2	203.6	2.044
**V**_f_	3.011	1747.35	76.11	216.02	2.957
**V**_g_	2.191	1456.59	83.51	128.59	5.53
**V**_h_	2.589	1233.72	83.87	451.84	4.027

*MTT assay*

Cell viability was assessed by MTT assay (microculture tetrazolium/formazan assay). After 24 h the medium of the cells which were seeded in 96 well plates was replaced by fresh medium containing compounds, each experiment was done in triplicate and six concentrations (20, 21). Tamoxifen was used as positive control. Then, the cells were incubated at 37°C for 72 h, the medium was changed by fresh medium containing MTT ([3-(4, 5- dimethylthiazol-2-yl)-2,4-diphenyltetrazolium bromide]) with a final concentration of 0.5 mg/mL. The cells were incubated for another 4 h in a humidified atmosphere at 37°C and after that the medium containing MTT was removed and remaining MTT formazan crystals were dissolved in 200 μL DMSO. The absorbance was measured at 570 nm after 20 min shaking using an ELISA reader. IC_50_ was defined as the concentration of the compounds that produced a 50% decrease in cell viability relative to the negative control which was wells exposed to the solvent without any compound. IC50 was determined by plotting a graph of Log (concentration of compound) *vs *% effect (cell viability). A line drawn from 50 % value on the *Y *axis meets the curve and interpolate to the *X *axis. The X axis value gives the Log (concentration of compound). The antilog of that value gives the IC50 value.

**Figure 6:**
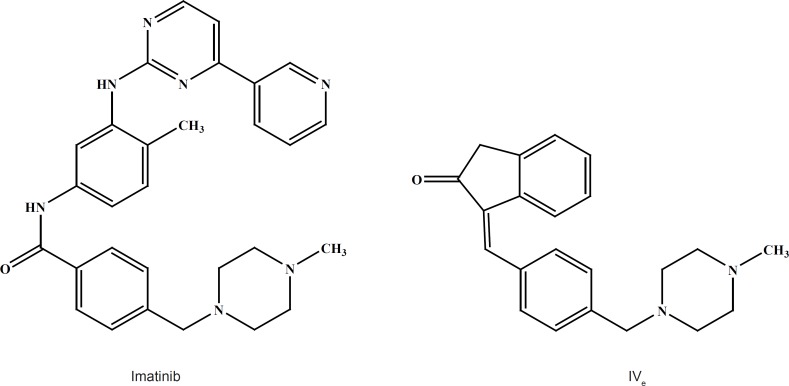
chemical structures of Imatinib and compound IV_e_

## Results and Discussion

All the synthesized compounds were assayed for their cytotoxic activities against colon cancer cell lines (HT29) and breast cancer cell lines (MCF7). The results are presented in Table 2.

As it appears from Table 2, the most active compounds are among the 3-benzylidene indole-2-one derivatives (IVseries). In 3-phenyliminoindole-2-ones (V series) only compound V_g_ showed moderate cytotoxic activity (27.2 µM against MCF7 and 61.9 µM against HT29). Compounds IV_a_ and IV_b _in 3-benzylidene indole-2-one series showed the best activities against both MCF7and HT29 cell lines. The most potent compound against MCF7 breast cancer cell is compound IV_a_ which bears 5-bromo substitution. This is in complete agreement with the report by Olgen et al for a series of N-benzyl-indole derivatives. They have found the 5-bromo substituted derivatives as the most potent inhibitors of c-Src tyrosine kinase.

 Compound IV_d _which shows highest activity against HT29 (7.51 µM) has a moderate activity (42.07 µM) against MCF7.

Compound IV_d_ is a 3-benzylidene indole-2-one derivative with a methyl piperazine moiety similar to the structure of Imatinib which is a 2-phenylaminopyrimidine derivative that functions as a specific inhibitor of a number of tyrosine kinase enzymes (Figure 6).

Global physicochemical properties for compounds IV_a-e_ and V_a-h _were calculated using Chemdraw Ultra version 8.0 and the results are presented in Table 3.

Efforts to find a relationship between these physicochemical parameters and cytotoxic activities of the compounds did not result in a clear correlation. The only interesting point in this regard is the closeness of CLogP values for the two most active compounds IV_a _and IV_b_ which are 3.7836 and 3.7808 respectively.

Studies are in progress to examine whether further structural modifications, specialy with regard to substituents at the 5 position, can result in enhancement of the cytotoxic activity of 3-benzylidene indole-2-one derivatives. Since MTT assay is a general screening method to evaluate the cytotoxicity of compounds and lacks specificity for anti-cancer evaluation, further mechanistic studies are needed to demonstrate the anti cancer and anti apaptosis activity of these compounds.

Src family of thyrosine kinases and other members of kinase enzymes which are proven to be involved in human colon and/or breast cancer are among the best candidates for the future enzyme inhibition studies.
